# Long-term results of intensity-modulated radiotherapy with three dose-fractionation regimens for localized prostate cancer

**DOI:** 10.1093/jrr/rry089

**Published:** 2018-12-19

**Authors:** Shinya Takemoto, Yuta Shibamoto, Chikao Sugie, Yoshihiko Manabe, Takeshi Yanagi, Hiromitsu Iwata, Taro Murai, Satoshi Ishikura

**Affiliations:** 1 Department of Radiology, Nagoya City University Graduate School of Medical Sciences, 1 Kawasumi, Mizuho-cho, Mizuho-ku, Nagoya, Aichi, Japan; 2 Department of Radiology, Fujieda Heisei Memorial Hospital, 123-1 Mizukami, Fujieda, Shizuoka, Japan; 3 Department of Radiation Oncology, Nanbu Tokushukai Hospital, 171-1 Hokama, Yaese-cho, Shimajiri-gun, Okinawa, Japan; 4 Department of Radiology, Narita Memorial Hospital, 134 Haneihonmachi, Toyohashi, Aichi, Japan; 5 Department of Radiation Oncology, Nagoya Proton Therapy Center, Nagoya City West Medical Center, 1–1-1 Hirate-cho, Kita-ku, Nagoya, Aichi, Japan

**Keywords:** prostate cancer, intensity-modulated radiotherapy, long-term outcomes, late toxicity

## Abstract

We evaluated long-term outcomes of three protocols of intensity-modulated radiation therapy (IMRT) for localized prostate cancer. Between 2005 and 2014, 348 patients were treated with 5-field IMRT. The first 74 patients were treated with a daily fraction of 2.0 Gy to 74 Gy (low-risk prostate cancer) or 78 Gy (intermediate- or high-risk prostate cancer); then 101 patients were treated with 2.1-Gy daily fractions to 73.5 or 77.7 Gy. More recently, 173 patients were treated with 2.2-Gy fractions to 72.6 or 74.8 Gy. The median age of all patients was 70 years and the median follow-up period was 82 months. The median follow-up periods were 124 months in the 2.0-Gy group, 98 months in the 2.1-Gy group, and 69 months in the 2.2-Gy group. The overall and prostate-specific antigen (PSA) failure-free survival (PSA-FFS) rates were, respectively, 89 and 68% at 10 years for the 2.0-Gy group, 91 and 84% at 8 years for the 2.1-Gy group, and 93 and 92% at 6 years for the 2.2-Gy group. The PSA-FFS rate for high-risk patients in all groups was 80% at 7 years. The cumulative incidences of Grade ≥2 late genitourinary (GU) and gastrointestinal (GI) toxicity were, respectively, 7.2 and 12.4% at 10 years for the 2.0-Gy group, 7.4 and 14.1% at 8 years for the 2.1-Gy group, and 7.1 and 7.9% at 6 years for the 2.2-Gy group. All three fractionation schedules yielded good tumor control with acceptable toxicities.

## INTRODUCTION

Intensity-modulated radiation therapy (IMRT) has been fairly well established as a definitive treatment for prostate cancer in Japanese patients. Accordingly, the proportion of patients undergoing radiation therapy as an initial therapy has increased in Japan [1]. Another study reported that definitive IMRT using helical tomotherapy appeared to be a valuable treatment option for patients with localized and locally advanced prostate cancer, even in extremely elderly patients [2]. However, IMRT with conventional regimens (1.8–2 Gy per daily fraction) takes nearly 2 months or longer, and this long treatment period may be disadvantageous compared with brachytherapy and recently developed stereotactic body radiotherapy. Several studies have suggested a low α/β ratio for prostate adenocarcinoma (1–1.8 Gy) [3–5], even lower than the ratios of late-responding normal tissues such as the bladder and rectum [6, 7]. Therefore, shorter treatment periods using a higher dose per fraction would be expected to improve therapeutic outcomes and make IMRT economically attractive [8].

Due to its relatively short history, reports of long-term results of IMRT for prostate cancer in the Japanese population are fewer than those in Western populations. In our institution, we started IMRT with 2.0-Gy daily fractions for localized prostate cancer using five static beams. Thereafter, the number of patients waiting for the treatment steadily increased because the 2.0-Gy daily fractionation schedule took a long time (~8 weeks). So, shortening the overall treatment time was desirable. At that time, however, the existing data were relatively short-term and insufficient to support the safety and usefulness of hypofractionated regimens, so we attempted to increase the daily dose in a step-by-step manner. After evaluating middle-term toxicities of the 2.0-Gy regimen, we investigated a 2.1-Gy daily fractionation schedule and then a 2.2-Gy regimen to shorten the treatment period in a stepwise fashion. After increasing the dose to 2.2 Gy per day, the protocol was continued until recently, when the number of the patients became stable due to the introduction of IMRT in nearby facilities.

In 2012, tomotherapy was introduced, and volumetric-modulated arc therapy also became available from 2015; so, 5-field IMRT is no longer used at our institution. Moreover, we have used a 2.5-Gy daily fraction since April 2018, because the use of hypofractionated regimens has become the worldwide trend based on the favorable clinical results of hypofractionation [9–14] and higher medical fees are now allocated to the hypofractionation schedule (≥2.5 Gy/day) in Japan. Therefore, the purpose of this study was to evaluate the long-term clinical outcomes of 5-field IMRT for localized prostate cancer and our three dose-fractionation regimens by updating the results reported in our previous publication [15]. We included new patients, extended follow-up periods from the previous study, and evaluated 10-year results for the 2.0-Gy protocol, 8-year results for the 2.1-Gy protocol and 6-year results for the 2.2-Gy protocol in this study.

## MATERIALS AND METHODS

### Patient characteristics

This was a retrospective study of protocol-based treatments; 348 patients with biopsy-confirmed prostate cancer treated with a 5-field IMRT technique at Nagoya City University Hospital between January 2005 and June 2014 were analyzed. Protocols were approved by the institutional review board (No. 273), and written informed consent was obtained from all patients. Dose-fractionation protocols were revised twice, as reported in our previous study [15]. The first 74 patients were treated with a daily fraction of 2.0 Gy to a total of 74 Gy (low-risk prostate cancer) or 78 Gy (intermediate- or high-risk prostate cancer), and then 101 patients were treated with 2.1-Gy daily fractions to 73.5 or 77.7 Gy. More recently, 173 patients were treated with 2.2-Gy fractions to 72.6 or 74.8 Gy. The median age of all patients was 70 years (range, 54–83) and the median follow-up period was 82 months (range, 18–157). The median follow-up periods were 124 months in the 2.0-Gy group, 98 months in the 2.1-Gy group, and 69 months in the 2.2-Gy group. The patient characteristics are summarized in Table 1. The average age of the 2.0-Gy group was slightly lower (*P* = 0.01) and the 2.2-Gy group had a lower rate of low-risk patients than the other groups (*P* < 0.01). All patients were staged according to the 7th edition of TNM staging and D’Amico Risk Categories [16], using computed tomography (CT), magnetic resonance imaging (MRI) and bone scintigraphy.

**Table 1.** Patient characteristics

Group	All patients	2.0 Gy/day	2.1 Gy/day	2.2 Gy/day	*P*
Total dose (Gy)	72.6–74/74.8–78^a^	74/78^a^	73.5/77.7^a^	72.6/74.8^a^	
No. of patients	348	74	101	173	
Age (years)	54–83	54–80	56–80	56–83	0.01^b^
(median)	70	68	70	70	
Initial PSA (ng/ml)	2.6–283	3.2–283	4.6–241	2.6–248	0.95^b^
(median)	11.3	11.1	11.5	10.8	
Risk Low/intermediate/high	27/92/156	10/20/44	14/31/56	6/64/103	0.01^c^
T stage 1/2/3	81/114/80	24/30/20	27/43/31	40/81/52	0.64^c^
ADT	240 (87%)	51 (69%)	91 (90%)	171 (99%)	<0.01^c^
Use of anticoagulant	49 (18%)	15 (20%)	15 (15%)	29 (17%)	0.63^c^
Coexistent DM	47 (17%)	16 (22%)	11 (11%)	27 (16%)	0.15^c^
Follow-up (months)	18–157	25–157	20–123	18–94	
(median)	82	124	98	69	

PSA = prostate-specific antigen, ADT = androgen deprivation therapy, DM = diabetes mellitus. ^a^For low-risk/intermediate- or high-risk patients. ^b^Examined by one-factor analysis of variance. ^c^Examined by chi-squared test.

### IMRT and androgen deprivation therapy

We reported details of the IMRT methods in our previous studies [15, 17]. Patients were immobilized in a supine position with a whole-body vacuum bag system, and the CT scans were performed at 3.2-mm and reconstructed to 2.5-mm thickness. The contouring was completed by reference to MRI images. The clinical target volume included the entire prostate and seminal vesicles depending on the T stage of the patient. The dose constraints, including the dose to the rectum and bladder, for all groups are provided in detail in our previous publication [15]. Patients were treated with 18-MV X-rays from five static ports using an optically guided 3D-ultrasound target localization system.

Generally, neoadjuvant androgen deprivation therapy (ADT) was used for 6 months in intermediate- or high-risk patients, and adjuvant ADT for 2–3 years in high-risk patients. In the 2.0-Gy group, the proportion of patients undergoing ADT was lower (69%) than in the other groups (90 and 98% in the 2.1-Gy and 2.2-Gy groups, respectively, *P* < 0.01).

### Follow-up and data collection

We performed follow-up evaluations at 1- to 3-month intervals until 1 year, and every 3–6 months thereafter. All end points were calculated from the start of IMRT. Prostate-specific antigen (PSA) failure was defined as a PSA rise of ≥2 ng/ml above the nadir according to the Phoenix definition [18]. One patient who developed radiographic evidence of bone metastasis at low PSA levels was counted as a PSA-failure case. Toxicities were evaluated with the Common Terminology Criteria for Adverse Events version 4.0. Late toxicities were defined as those occurring later than 3 months after starting IMRT. In the present study, the toxicities for some of the patients were re-evaluated, so the toxicity data in the 2.0-Gy and 2.1-Gy groups is slightly different from those reported previously [15].

### Statistical analysis

Differences in patient characteristics and incidences of acute genitourinary (GU)/gastrointestinal (GI) toxicities between groups were examined by one-factor analysis of variance and the chi-squared test. Overall survival rates, PSA-failure-free survival (PSA-FFS) rates and cumulative incidences of Grade ≥2 late toxicity were calculated by the Kaplan–Meier method, and differences between groups were examined by the log-rank test. We used univariate and multivariate Cox proportional hazards model to investigate the associations of patient characteristics (age, risk classification, ADT, use of anticoagulants and presence of diabetes mellitus) with outcomes and with toxicities. All statistical analyses were performed with EZR (Saitama Medical Center, Jichi Medical University, Saitama, Japan), which is a graphical user interface for R (The R Foundation for Statistical Computing, Vienna, Austria). More precisely, it is a modified version of R commander designed to add statistical functions frequently used in biostatistics [19].

## RESULTS

**Fig. 1.**
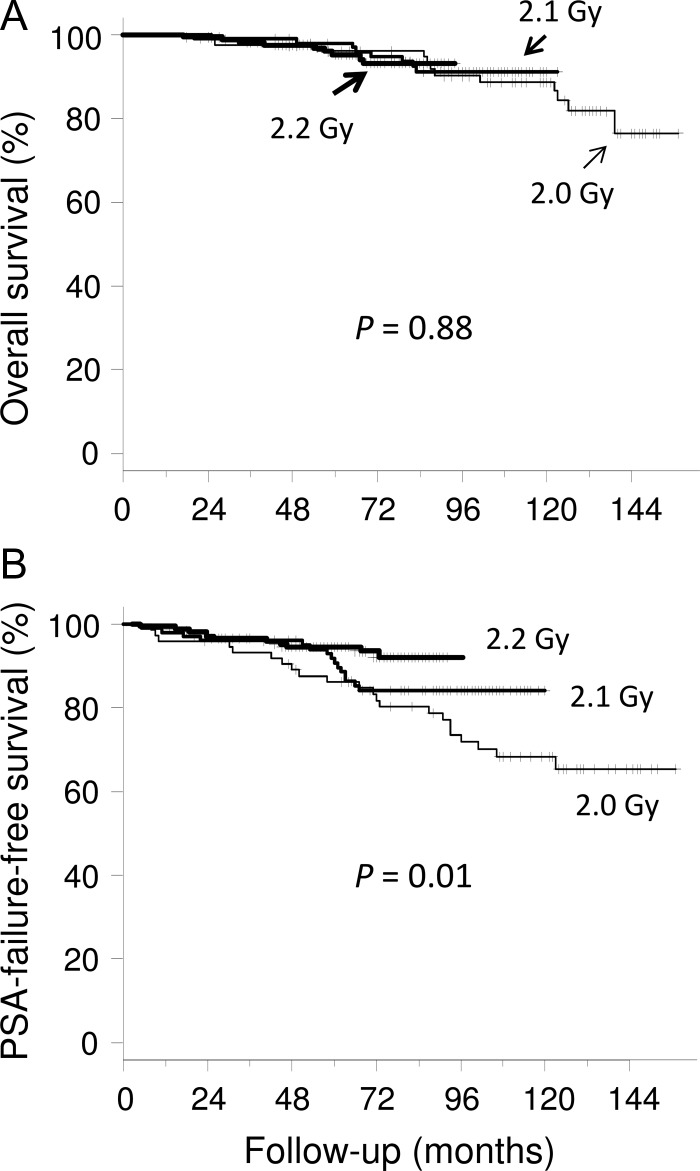
Overall and prostate-specific antigen (PSA)-failure-free survival curves for the three dose groups.

**Fig. 2.**
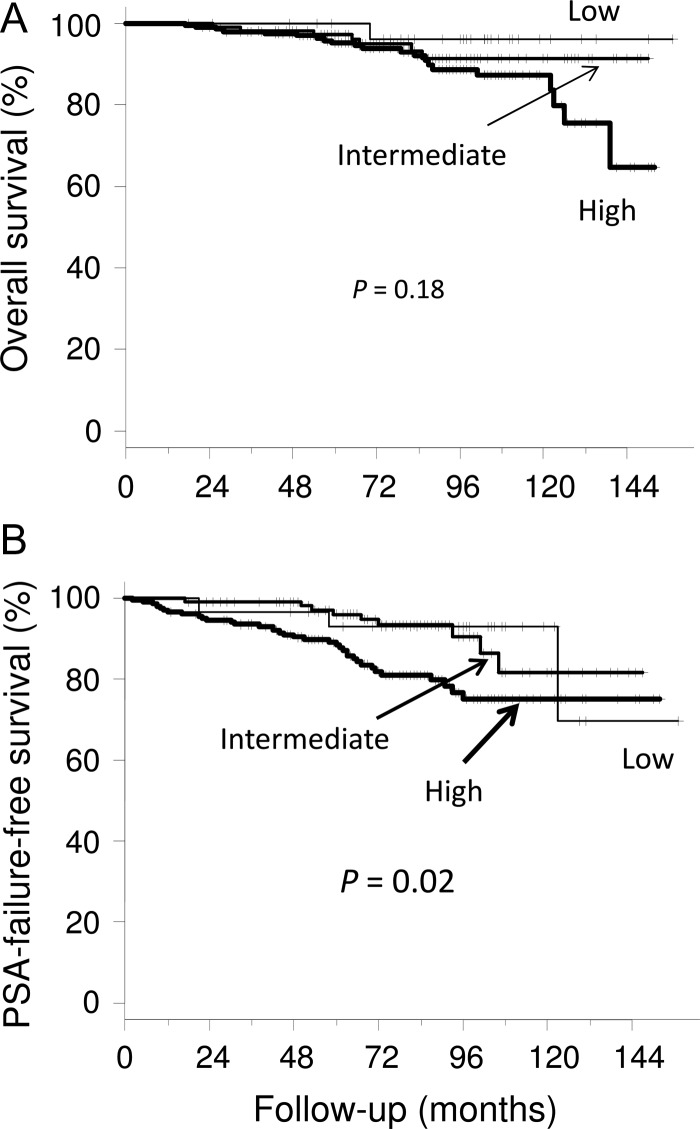
Overall and prostate-specific antigen (PSA)-failure-free survival curves for the three risk groups.

Overall survival and PSA-FFS rates were, respectively, 89 and 68% at 10 years for the 2.0-Gy group, 91 and 84% at 8 years for the 2.1-Gy group, and 93 and 92% at 6 years for the 2.2-Gy group (Fig. 1). Six patients died of prostate cancer and 23 patients died from intercurrent diseases. No significant difference was found in overall survival between the three dose groups (*P* = 0.88), but a difference was found in PSA-FFS (*P* = 0.01). When comparing pairs of dose groups, there was a difference only between the 2.0-Gy and 2.2-Gy groups (*P* = 0.02). No significant differences were found in overall survival between the three risk groups (*P* = 0.18) (Fig. 2). The PSA-FFS rate for high-risk patients in all groups was 80% at 7 years, while the rate was 93% for both intermediate- and low-risk patients (*P* = 0.02 for the three groups). Three low-risk patients (10%) had PSA failure, and one of them relapsed at 123 months.

The incidences of Grade 2 acute GU and GI toxicity were, respectively, 9.5 and 1.4% for the 2.0-Gy group, 19.8 and 2.0% for the 2.1-Gy group, and 20.8 and 1.2% for the 2.2-Gy group (*P* = 0.09 and 0.74, respectively). No Grade ≥3 acute toxicity was observed. The cumulative incidences of Grade ≥2 late GU and GI toxicity were, respectively, 7.2 and 12.4% at 10 years for the 2.0-Gy group, 7.4 and 14.1% at 8 years for the 2.1-Gy group, and 7.1 and 7.9% at 6 years for the 2.2-Gy group (*P* = 0.98 and 0.29, respectively) (Table 2). Four (1.1% of all patients) developed Grade 3 GU toxicities at 28, 41, 53 and 64 months, respectively: two developed severe hematuria requiring endoscopic coagulation and blood infusion, one each developed urinary retention, and one developed hydronephrosis. Improvement was achieved by conservative treatment including urethral catheterization. Argon plasma coagulation (APC) was administered to 16 patients (4.6% of all patients) for rectal hemorrhage. Three patients (0.9% of all patients) had Grade 3 GI toxicity: patient A at 12 months, patient B at 27 months and patient C at 28 months, respectively. Blood transfusion and APC were performed. No patients had Grade ≥4 toxicity.

**Table 2.** Grade ≥2 late toxicities

	Grade 2/3 toxicity			*P*
	2.0 Gy/day	2.1 Gy/day	2.2 Gy/day	
Genitourinary				
Urinary frequency	1/0	1/0	5/0	
Hematuria	2/0	2/2	0/1	
Urinary retention	0/1	2/0	1/0	
Urinary incontinence	1/0	0/0	4/0	
Total^a^	7.2%^b^	7.4%^c^	7.1%^d^	0.98^e^
Gastrointestinal				
Rectal hemorrhage	9/0	11/3	12/0	
Total^a^	12.4%^b^	14.1%^c^	7.9%^d^	0.29^e^

^a^Cumulative incidence of Grade ≥2 late genitourinary/gastrointestinal toxicity. ^b^Incidence at 10 years. ^c^Incidence at 8 years. ^d^Incidence at 6 years. ^e^Examined by logrank test.

On univariate analysis, age ≥70 years was associated with worse overall survival (*P* = 0.01), and high risk was associated with worse PSA-FFS (*P* = 0.006). Use of anticoagulants was associated with Grade ≥2 late GI toxicity (*P* = 0.03). Table 3 shows results of multivariate analyses for overall survival, PSA-FFS and late Grade ≥2 toxicities. The use of anticoagulants became insignificant for Grade ≥2 late GI toxicity (*P* = 0.06). The presence of diabetes mellitus was not a significant factor for Grade ≥2 late GU or GI toxicity.

**Table 3.** Multivariate analyses for overall survival, PSA-FFS, Grade ≥2 late GU and GI toxicity

	Overall survival		PSA-FFS		Late GU toxicity		Late GI toxicity	
	Hazard ratio	*P*	Hazard ratio	*P*	Hazard ratio	*P*	Hazard ratio	*P*
Age	2.73	0.02	0.89	0.68	1.90	0.17	1.27	0.49
(≥ vs <70 years)	(1.19–6.27)		(0.50–1.57)		(1.77–4.71)		(0.65–2.50)	
Risk	1.62	0.26	2.66	0.005	1.09	0.86	1.15	0.71
(HR vs LR/IR)	(0.70–3.72)		(1.35–5.22)		(0.43–2.70)		(0.56–2.34)	
ADT	3.22	0.13	1.12	0.81	1.32	0.72	4.33	0.15
(Yes vs no)	(0.71–14.51)		(0.43–2.96)		(0.30–5.81)		(0.59–32.00)	
Anticoagulant	1.66	0.23	0.51	0.13	1.62	0.33	2.03	0.06
(Yes vs no)	(0.73–3.81)		(0.21–1.22)		(0.61–4.26)		(0.97–4.24)	
Coexistent DM	0.86	0.76	1.43	0.32	1.42	0.49	1.71	0.17
(Yes vs no)	(0.32–2.29)		(0.71–2.90)		(0.52–3.92)		(0.80–3.66)	

LR, IR, HR = low, intermediate, and high risk, ADT = androgen deprivation therapy, DM = diabetes mellitus.

## DISCUSSION

The three dose fractionation regimens yielded similar results in overall survival and toxicities, but the 2.2-Gy group had a higher PSA-FFS rate than the 2.0-Gy group. There were differences in the follow-up duration: the median follow-up period for the 2.2-Gy group was 69 months, but several patients in the 2.0-Gy group developed PSA failure after 6 years. The proportion of patients undergoing ADT was lower in the 2.0-Gy group; this was in part due to the higher proportion of low-risk patients; in addition, a proportion of the intermediate- and high-risk patients in the 2.0-Gy group did not undergo ADT. Also, a learning curve effect might have developed in our treatment planning and actual treatment, which would lead to better outcomes in more recent patients (i.e. the 2.2-Gy group patients). These facts may account for the difference in the PSA-FFS rate. Further investigations are necessary to evaluate whether shortening the treatment period contributes to therapeutic gains. Nevertheless, all groups obtained favorable overall survival and PSA-FFS rates.

The rates of acute Grade ≥2 GU toxicities (9.5–20.8%) and late Grade 3 GU toxicities (1.1%) for all patients in this study seemed to be comparable with or compare favorably with those in previous studies [9, 10, 12, 20]. Arcangeli *et al.* [21] reported that urinary toxicity continued to increase beyond 4 years, whereas rectal toxicity plateaued at 20–26 months. In this study, the cumulative incidence of Grade ≥2 GU toxicities in the three dose groups increased until 66–72 months. Longer follow-up is necessary to evaluate late Grade ≥2 GU toxicity. Grade ≥2 late GI toxicities (10.8% at 7 years in all patients) were also comparable with those reported in previous studies [20, 22]. As we previously reported, our treatment strategy and outcome for patients with late rectal bleeding mean that this adverse event may not be so troublesome [23]. Some risk factors such as the use of anticoagulants, diabetes mellitus and the high-dose-irradiated volume of the rectum have been reported [24–27]. In this study, the use of anticoagulants and diabetes mellitus were not significant risk factors for late Grade ≥2 GI toxicity in multivariate analysis. This might have been due to the relatively small patient number.

Several Japanese groups have reported long-term outcomes of conventionally fractionated regimens [28–30]. Hypofractionated regimens have also been studied to increase therapeutic gain. A few institutions have reported 5-year or longer outcomes of slightly or moderately hypofractionated regimens using 2.2–3-Gy daily fractions with satisfactory disease controls and acceptable toxicities [31, 32]. Hypofractionated radiation therapy regimens have recently been compared with conventional radiation therapy in randomized trials [9–13]. According to a systematic review and meta-analysis of these trials [14], biochemical failure, biochemical and/or clinical failure, overall mortality, prostate cancer–specific mortality, acute GU toxicity, and late GU and GI toxicities were all similar. Nevertheless, the incidence of acute GI toxicity was 9.1% lower with the conventional regimen. Several studies in Japan have reported toxicities of hypofractionated regimens, with high incidences of late rectal toxicities observed in some of the studies [33–35]. Further investigations are necessary to determine the optimal fractionation schedule.

Optimal daily and total doses should be determined carefully. The LQ model is often used to estimate the equivalence between different fractionation regimens, but it does not take reoxygenation into consideration. Recent laboratory studies suggest that the LQ model overestimates the effect of a high fractional dose of radiation [36–38]. Since the α/β ratio represents the dose at which cell killing from linear (α) and quadratic (β) components of the LQ formula is equal, this model is considered appropriate when used for daily doses around the α/β ratio [36]. With increase in the fractional doses, however, the β cell kill component dominates in the LQ model; thus, actual data would deviate. Hence, it was proposed that the LQ model might only be applicable for fractional doses up to twice the α/β ratio [36]. Based on these considerations, our approach for stepwise shortening of the overall treatment time based on the LQ model seems reasonable, and so far it has yielded the expected outcomes. We are now using a daily dose of 2.5 Gy. In this study, we reported the culmination of our 2.0–2.2-Gy daily fractionation regimens.

Our study has a few limitations. This was not a well-controlled study, and there were imbalances between the three groups in terms of patient characteristics, especially risk-group distributions, proportions of patients undergoing hormone therapy, and follow-up periods. Comparison between the three dose-fractionation groups might reflect these biases, and may not be so useful. In future, randomized studies on the optimal fractionation schedules are warranted.

In conclusion, tumor control was good and toxicities were acceptable in all dose groups after long-term follow-up periods, suggesting that our stepwise shortening of treatment periods has been successful.
